# The Use of Digital Technologies, Impulsivity and Psychopathological Symptoms in Adolescence

**DOI:** 10.3390/bs9080082

**Published:** 2019-07-24

**Authors:** Luca Cerniglia, Marco Guicciardi, Maria Sinatra, Lucia Monacis, Alessandra Simonelli, Silvia Cimino

**Affiliations:** 1 Faculty of Psychology, International Telematic University Uninettuno, 00186 Rome, Italy; 2 Department of Education, Psychology, Philosophy, University of Cagliari, 09123 Cagliari, Italy; 3 Department of Educational Science, Psychology, Communication, University of Bari, 70122 Bari, Italy; 4 Department of Humanities, University of Foggia, 71121 Foggia, Italy; 5 Department of Developmental Psychology and Socialisation, Faculty of Psychology, University of Padua, 35131 Padova, Italy; 6 Department of Dynamic and Clinical Psychology, Sapienza University of Rome, 00185 Rome, Italy

**Keywords:** internet gaming disorder, gaming addiction, social media addiction, online addictions, impulsivity, psychopathology

## Abstract

*Background and aims:* Past research on the associations between psychopathological symptoms and technological-based addictions, i.e., Internet Gaming Disorder (IGD) and Social Media Addiction (SMA), showed contradictory results in adolescents and adult populations. The present study investigated correlations between adolescents’ psychopathological risks and impulsivity, IGD and SMA. *Methods:* A sample of 656 participants (338 males; M_age_ = 16.32 years) was divided into three age groups (early, mid-, and late adolescence) and completed a battery of scales comprising the (i) Internet Gaming Disorder Scale–Short Form, (ii) Bergen Social Media Addiction Scale, (iii) Barratt Impulsiveness Scale for Adolescents, and (iv) Symptom Checklist-90-R. *Results:* The significance of the correlations was not corroborated in the basic tables. Significant associations appeared only in the adolescent subgroups, sometimes for bivariate and sometimes for partial correlations and with different patterns of associations between males and females. Moreover, both technological addictions were correlated with impulsiveness in bivariate and partial correlations. *Discussion and conclusions:* Following a developmentally-oriented approach to determine the patterns of associations between technological behavioral addictions and psychopathology in the specific sub-phases of early-, mid- and late-adolescence, this exploratory research showed how these associations might change depending on the developmental phase and gender of the individual. Future research is needed to provide empirical evidence of specific emotional–psychopathological correlations.

## 1. Introduction

Despite the general exciting view of technology in various fields, from education to health, researchers have recently highlighted the risks of technology whose overuse could lead to mental disorders [1]. 

In this regard, Internet Gaming Disorder (IGD), defined as a behavioral pattern encompassing persistent and recurrent use of the Internet to engage in games, has been recently included in Section III of the fifth edition of the DSM-5 (American Psychiatric Association, 2013) [2], leading to an increased interest of researchers to foster a consensual view of this phenomenon, given the various theoretical models of technology-based addictions [3]. However, the same DSM-5 has stressed the need to provide further empirical evidence to legitimize IGD as a separate mental disorder and to determine the diagnostic validity of each criterion. It is noteworthy to mention that the World Health Organization (WHO, 2017) [4] has recently included Gaming Disorder in the beta version of the ICD-11 (International Classification of Diseases), defining the problem as a pattern of persistent or recurrent gaming behavior (‘digital gaming’ or ‘video-gaming’), which may be online (i.e., over the Internet) or offline.

The nine IGD clinical diagnostic criteria have been borrowed from gambling disorder or substance use disorder and directly map onto the six criteria of Griffiths’ symptom-centered model of addiction based on a biopsychological perspective. The model has been widely applied to the conceptualization of many technology-based addictions, such as IGD and Social Media Addiction (SMA), although the latter has no status in the DSM-5 yet [5,6].

The six criteria refer to: mood modification (engagement in gaming or Social Network Sites [SNSs] leading to a favorable change in emotional states), salience (behavioral, cognitive, and emotional preoccupation with the games or SNS usage), tolerance (ever increasing use of games or SNSs over time), withdrawal symptoms (experiencing unpleasant physical and emotional symptoms when gaming or SNS use is restricted or stopped), conflict (interpersonal and intrapsychic problems ensuing because of gaming or SNS usage), and relapse (addicts quickly reverting back to their excessive gaming or SNS usage after a period of abstinence).

Although past research on risk factors linked to IGD and SMA showed associations between both technological addictions and attention-deficit/hyperactivity disorder, obsessive-compulsive disorder [7], depressive symptoms [8,9], mood and anxiety symptoms [10], González-Bueso and colleagues(2018) have stressed the inconsistent nature of the results [11]. For instance, previous literature has found correlations in adults, whereas low and very low associations have been found in adolescents [12]. However, in the complex developmental stage of adolescence, teenagers appear to show a specific vulnerability to a broad range of psychopathological symptoms, both in the internalizing (e.g., depression, anxiety, somatization) and externalizing area (e.g., hostility). All these symptoms should be evaluated when studying these populations. With regards to the use of new technologies, social networks and the internet, adolescents tend to show problematic use of the internet, which is probably due to the immaturity of the cerebral cortex and to an imbalance between the maturation of different regions, whose consequence is an affective and behavioral dysregulation [13,14]. In fact, this dysregulation could be at the root of adolescents’ impulsivity, which in turn, has been found to be one of the most predictive personality factors of IGD and SMA, especially in adolescent and emerging adult populations. Therefore, the assessment of impulsivity levels in these samples is important and useful to understand the links among the variables [15,16,17]. The few studies focusing on adolescents in this specific field have recruited participants in a very narrow range of age (mainly from 14 to 16 years of age) and there is a lack of research considering samples representing the different stages of adolescence (early, mid- and late adolescence).

Without eliciting the directional relationships, such exploratory research could shed light on the possible comorbidity between maladaptive psychological functioning, impulsivity, and behavioral addictions in specific developmental phases.

The research question of this exploratory study was to investigate correlations between adolescents’ psychopathological risks (somatization, obsessive-compulsive, interpersonal sensitivity, depression, anxiety, hostility, phobic anxiety, paranoid ideation, psychoticism), impulsivity, and two technology-based addictions, i.e., IGD and SMA. Gender was also included as a socio-anagraphic risk factor, following previous findings [18]. Indeed, males and females have been found to have different characteristics in their internet gaming and social networks use, as well as in their impulsivity levels and psychopathological risk [19]. Chen and colleagues [20] have shown males’ higher likelihood of engaging in internet games, whereas Bekhbat and Neigh [21] have confirmed females’ higher vulnerability to depression.

## 2. Method

### 2.1. Participants and Procedure

A sample of 656 participants (338 males; M_age_ = 16.32 years, SD = 1.54), recruited from high schools in the regions of Central Italy, and divided into three age groups: early adolescence (14–15 years; 40.3% of the entire sample), mid-adolescence (16–17 years; 39.2% of the entire sample), and late adolescence (18–19 years; 20.5% of the entire sample). Participants were invited to complete an online self-report questionnaire, which took approximately 50 min to complete. Data collection took place from March to May 2017 during school hours. The procedure was agreed among researchers, headmasters and teachers of the schools. One trained researcher, blind to the scope of the study, was present during the sessions to explain the procedure and answer students’ questions about the meaning of the items. One teacher was present, but he/she had been indicated not to talk to students and/or suggest answers. Each student filled out a computerized version of the questionnaires. Social desirability was controlled through lie items, as per the authors’ recommendation.

### 2.2. Measures

The Internet Gaming Disorder Scale–Short Form (IGDS9-SF) [22,23] is a unidimensional tool that consists of 9 items assessing the nine core criteria for IGD defined by DSM-5 by analyzing both online and/or offline gaming activities occurring over a 12-month period. Each item is answered on a 5-point Likert scale ranging from 1 (*Never*) to 5 (*Very often*). Examples of items include: “*Do you feel the need to spend increasing amount of time engaged gaming in order to achieve satisfaction or pleasure?*” and “*Have you continued your gaming activity despite knowing it was causing problems between you and other people?*”. Higher scores mean a higher degree of gaming disorder. Internal reliability for the IGDS9-SF for the sample in the present study was found to be very good (Cronbach’s α = 0.88) in line with the values reported in other international research [24].

The Bergen Social Media Addiction Scale (BSMAS) [7,25] assesses the experience of using social media within a 12-month period. It comprises six items rated on a 5-point Likert scale from 1 (*Very rarely* to 5 = *Very often*) and related to core addiction elements (salience, mood modification, tolerance, withdrawal, conflict, and relapse). Examples of items include: “*How often during the last year have you used social media so much that it has had a negative impact on your job/studies?*” and “*How often during the last year have you felt an urge to use social media more and more?*”. A higher score of the scale means stronger addiction to the social media, and a score over 19 means that an individual is at-risk of developing problematic social media use. In the current research, the scale obtained a good internal consistency (Cronbach’s α = 0.78).

The Barratt Impulsiveness Scale for Adolescents (BIS-11-A) [26,27,28] is a 30-item self-applied measure commonly used for the assessment of impulsiveness in research as well as in clinical settings. The items provide a total score and three sub-scores: attention, lack of planning, motor impulsivity. Each item is rated on a 4-point Likert scale ranging from 1 (Never) to 4 (*Very often*). Examples of items are: “*I do things without thinking*” and “*I say things without thinking*”. Higher scores indicate higher levels of impulsiveness. In the present study, the overall impulsiveness score was calculated following the suggestions of Fossati et al. [28] (p. 632). The internal consistency of the scale was found to be good (Cronbach’s α = 0.78).

The Symptom Checklist-90-R (SCL-90-R) [29,30] is a widely used 90-item self-report inventory indicating the rate of occurrence of the symptom during the past seven days. It was developed to measure symptom intensity on nine different subscales: Somatization (SOM), Obsessive-Compulsive (O-C), Interpersonal Sensitivity (I-S), Depression (DEP), Anxiety (ANX), Hostility (HOS), Phobic Anxiety (PHOB), Paranoid Ideation (PAR), and Psychoticism (PSY). The items in the inventory are scored on a 5-point scale ranging from 1 (*Not at all*) to 5 (*Extremely*). In the current research, the subscales of the SCL90-R showed good to excellent internal consistencies (Cronbach’s α = 0.93 for SOM, 0.92 for O-C, 0.81 for I-S, 0.89 for DEP, 0.90 for ANX, 0.74 for HOS, 0.85 for PHOB, 0.88 for PAR, and 0.89 for PSY).

### 2.3. Statistical Analysis

Data analyses included descriptive statistics (means and standard deviations), zero-order and partial correlations between the variables of interest for the total sample and for each gender and age group.

### 2.4. Ethics

The research study complied with the general ethical principles of the Declaration of Helsinki and was approved by the research team’s University Institutional Review Board, approval number is 10/2018 issued by La Sapienza University of Rome. Permission was required from school heads to conduct the research. Written informed consent was obtained from students aged over 18 years and from parents or legal guardians for students aged under 18 years.

## 3. Results

Means and standard deviations of the raw item scores of the study variables are shown in Table 1. This sample does not exceed the clinical cut-offs indicated in previous literature in any of the considered variables (for norms and cut-off points, please see [23,24,28,29]).

### 3.1. Independent Sample t-Test and Univariate ANOVA

No significant sex and age differences emerged in all observed variables.

### 3.2. Bivariate Correlations

Pearson’s correlations were computed between the scores of IGD, SMA, impulsiveness, and psychopathological dimensions for the total sample, gender groups, and the three adolescent age groups. As for the total sample, results indicated that IGD was positively correlated with SMA and impulsiveness, and SMA was positively correlated with impulsiveness (Table 2). With regard to the gender groups, results showed positive correlations between IGD, SMA and impulsiveness, and between SMA and impulsiveness in both males and females. No associations emerged between psychopathological symptoms and technological addictions (Table 3).

Bivariate correlations were calculated for gender per age groups. Findings showed that: (i) in females aged 14–15 years, IGD was positively correlated with SMA and impulsiveness, and SMA was positively correlated with impulsiveness, whereas in males aged 14–15 years, impulsiveness was positively correlated with SMA and IGD; (ii) IGD, SMA, and impulsiveness were positively correlated in males and females aged 16–17 years, whereas in males aged 16–17 years, IGD was negatively correlated with somatization, obsessive-compulsive, interpersonal sensitivity, anxiety, hostility, and phobia; and (iii) IGD was positively correlated with SMA and impulsiveness in males aged 18–19 and only to impulsiveness in females aged 18–19 (Table 4).

### 3.3. Partial Correlations

Since the dimensions of the SCL-90 were highly correlated, partial correlations between each technological addiction, impulsiveness and each symptom were performed by controlling the effect of the other symptoms in the total sample, gender groups and the three age groups. As for the total sample, no correlations emerged between IGD, SMA and psychopathological symptoms (Table 5). With regard to gender groups, results showed that IGD was correlated positively with psychoticism, and negatively, although marginally (*p* =0.055), with somatization in males (Table 6).

Results from partial correlations for gender per age group indicated that IGD was correlated positively to interpersonal sensitivity in females aged 14–15 years and to depression in females aged 16–17 years, and negatively to hostility in males aged 14–15 years, interpersonal sensitivity in males aged 16–17 years, and somatization in males aged 18–19 years. Conversely, SMA proved to be negatively correlated to interpersonal sensitivity and hostility in females aged 16–17 years, and to depression in males aged 18–19 years (Table 7).

## 4. Discussion

The present study sought to examine associations between adolescent technology-based addictions (IGD and SMA), and psychopathological symptoms and impulsivity. Findings from bivariate correlations showed that IGD was correlated with high levels of SMA in all groups, and was, therefore, in line with previous studies [5,7,23,31,32,33]. Impulsivity was correlated with higher levels of IGD in all groups, and with SMA in the groups of young and mid-aged adolescents. These results are consistent with prior studies [6,34,35,36] in which greater impulsivity was reported to constitute a risk factor for pathological gaming. This is in accordance with the assumption that pathological video game use/social media use are impulse-control disorders, and may, therefore, be characterized by the failure to resist an impulse. This association between impulsivity and SMA was not confirmed in the 18–19 year age group. In the younger adolescent groups, the association could be due to the relative immaturity of prefrontal cortex (PFC) in early and mid-adolescents [37,38]. The PFC underpins the cognitive reasoning processes and the dampening of affect and emotion-driven behaviors and completes its maturation in the later years of adolescence and emergent adulthood [39]. In adolescents, emotion-driven behaviors have been posited to occur primarily in social interactions and especially, in activities connected with peer relationships (such as the use of social networking sites). Compared to the younger participants, adolescents aged 18–19 years may rely on reduced impulsivity (due to neural maturation) and, therefore, reported no pathological use of social networking in the present study. However, some participants in this older age group still showed pathological gaming probably because of the particular nature of gaming activities, which while a social activity for many [40,41], can be engaged in without social interaction if the player so desires.

With regard to the associations between technology-based addictions and psychopathological factors, no correlation emerged between the variables in the total sample. However, when looking at age subgroups, higher levels of IGD were correlated with higher levels of interpersonal sensitivity in early adolescence. From the perspective of developmental psychopathology [42,43], the positive correlation may be explained by the desire for increased independence, increased emotional distance between early adolescents and their parents, and a more intense focus on social interactions and friendships. During this developmental stage, feelings of inferiority in comparison to other individuals, discomfort during interpersonal interactions, low self-esteem, and lack of assertiveness may arise as specific manifestations of interpersonal sensitivity [44,45]. Thus, the overuse of videogames could be viewed as a strategy to escape from these negative feelings. Conversely, in mid-adolescence, negative correlations between IGD and somatization, obsessive-compulsive, interpersonal sensitivity, anxiety, and hostility were observed, indicating that adolescents aged 16-17 years may play videogames as a strategy to control such psychopathological symptoms.

Partial correlations were then run to avoid the possible collinearity effects among the SCL-90 subscales that have been shown to suffer from this limitation [30]. Results confirmed the absence of significant associations between IGD, SMA, psychopathological symptoms, and impulsivity in the total sample. Similar to the bivariate correlations, significant associations between the variables were found in age subgroups, although with some different results. Indeed, IGD and SMA were positively correlated with depressive symptoms in adolescents aged 16–17 years, whereas IGD was negatively correlated with somatization in individuals aged 18–19 years, and SMA was negatively correlated with interpersonal sensitivity and hostility in adolescents aged 16–17 years. Two aspects are noteworthy: first, the only positive partial correlation characterized depression as a maladaptive symptom correlated with technology-based addictions; second, these addictions had counterintuitive (i.e., negative) relationships with hostility and interpersonal sensitivity. Despite the descriptive nature of this study, it could be speculated that the positive correlation may demonstrate adolescents’ overuse of videogames and social media to increase levels of emotional activation [46], whereas the negative correlations may refer to youths’ desire to reduce their psychological discomfort associated with the difficulty of coping with interpersonal interactions in offline life. This strategy could dampen youths’ perceived psychological distress, so that their psychopathological symptoms could be rated as less problematic.

A closer look at partial correlations for gender per age groups among IGD, SMA, and psychopathological symptoms highlighted a still more complex scenario contrary to bivariate correlations in the same subgroups. For instance, problematic gaming in females aged 14–15 years correlated with interpersonal sensitivity, whereas depression in females aged 16–17 years correlated with social network overuse. Moreover, IGD proved to be negatively correlated with hostility in males aged 14–15 years, and with somatization in males aged 18–19 years. Finally, SMA correlated negatively with hostility and interpersonal sensitivity in females aged 16–17 years. Consequently, the data here suggest that the main psychopathological dimensions related to IGD and SMA are depression, interpersonal sensitivity, hostility, and somatization. However, these relationships are controversial and might change depending on the developmental phase (early, mid- and late adolescence) and gender of the individual [47,48,49].

There is no doubt that the results of the present study suffer from some limitations. First, the racial/ethnic homogeneity of the sample hinters the generalization of the results to a wider population. Second, since the psychometric tools used in the present study are typically self-reported questionnaires, there is an understandable concern that they have impacted on the validity of the conclusions that have been drawn.

The findings of the present study have a number of potentially useful implications. Research focusing on the variables of problematic internet gaming, social media use, and impulsivity in a non-clinical adolescent population has typically dealt with the identification of at-risk groups on the basis of age, gender, and other characteristics [50], thus overlooking more clinical aspects, such as adolescents’ psychopathological risks. Furthermore, this study applied a developmentally-oriented approach to determine the patterns of associations, following Steinberg’s suggestion [51] to focus on the specific sub-phases of early, mid- and late adolescence. It is well-known that the levels of IGD, SMA, and impulsiveness can assume different clinical meanings in younger or older adolescents and in youths who show different associated psychological problems. Future research in the field of internet gaming and social media use should assume a developmentally-oriented perspective, going beyond the mere consideration of problematic behavioral manifestations (IGD and SMA) and examining more closely their emotional–psychopathological correlations.

## Figures and Tables

**Table 1:** Descriptive statistics for the variables of interest (total sample, gender and each age group).

	Males (n = 338)	Females (n = 318)	Total Sample (n = 656)	14–15 Years (n = 264)	16–17 Years (n = 257)	18–19 Years (n = 135)
	Mean (SD)					
IGD	1.62 (0.723)	1.62 (0.774)	1.62 (0.748)	1.66 (0.763)	1.63 (0.757)	1.54 (0.699)
SMA	2.089 (0.844)	2.163 (0.836)	2.12 (0.840)	2.15 (0.856)	2.15 (0.865)	2.02 (0.756)
IMP	2.586 (0.365)	2.599 (0.418)	2.59 (0.391)	2.58 (0.394)	2.60 (0.395)	2.59 (0.382)
	**Symptoms of psychopathology**					
SOM	0.540 (0.721)	0.467 (0.689)	0.50 (0.71)	0.49 (0.71)	0.51 (0.68)	0.52 (0.76)
O-C	0.465 (0.685)	0.399 (0.652)	0.43 (0.67)	0.43 (0.68)	0.42 (0.64)	0.46 (0.71)
I-S	0.455 (0.558)	0.420 (0.534)	0.44 (0.55)	0.43 (0.54)	0.44 (0.53)	0.45 (0.59)
DEP	0.606 (0.695)	0.522 (0.704)	0.56 (0.70)	0.52 (0.68)	0.58 (0.67)	0.61 (0.79)
ANX	0.481 (0.672)	0.438 (0.650)	0.46 (0.66)	0.45 (0.67)	0.45 (0.63)	0.51 (0.72)
HOS	0.365 (0.523)	0.336 (0.510)	0.35 (0.52)	0.33 (0.51)	0.35 (0.50)	0.38 (0.57)
PHOB	0.429 (0.627)	0.389 (0.612)	0.41 (0.62)	0.41 (0.62)	0.40 (0.59)	0.43 (0.66)
PAR	0.417 (0.616)	0.370 (0.606)	0.39 (0.61)	0.36 (0.59)	0.42 (0.62)	0.41 (0.65)
PSY	0.447 (0.623)	0.453 (0.631)	0.45 (0.63)	0.44 (0.62)	0.44 (0.59)	0.48 (0.70)

IGD = Internet Gaming Disorder; SMA = Social Media Addiction; IMP = Impulsiveness; SOM = Somatization; O-C = Obsessive-Compulsive; I-S = Interpersonal Sensitivity; DEP = Depression; ANX = Anxiety; HOS = Hostility; PHOB = Phobic Anxiety; PAR = Paranoid Ideation; PSY = Psychoticism.

**Table 2:** Bivariate correlations between the variables of interest for the total sample.

	IGD	SMA	SOM	O-C	I-S	DEP	ANX	HOS	PHOB	PAR	PSY
IGD	-	0.287 **	−0.014	0.001	0.012	0.032	0.004	−0.009	0.012	0.000	0.030
SMA	-	-	−0.021	−0.006	−0.023	0.007	−0.009	−0.029	0.008	−0.011	0.002
IMP	0.287 **	0.306 **	−0.033	−0.032	−0.048	−0.039	−0.040	−0.052	−0.021	−0.022	−0.012

* *p* < 0.05; ** *p* < 0.01. SMA = Social Media Addiction; IGD = Internet Gaming Disorder; IMP = Impulsiveness; SOM = Somatization; O-C = Obsessive-Compulsive; I-S = Interpersonal Sensitivity; DEP = Depression; ANX = Anxiety; HOS = Hostility; PHOB = Phobic Anxiety; PAR = Paranoid Ideation; PSY = Psychoticism.

**Table 3:** Bivariate correlations between the variables of interest for each gender group.

	IGD	SMA	SOM	O-C	I-S	DEP	ANX	HOS	PHOB	PAR	PSY
**Females**											
IGD	-	0.331 **	0.013	0.026	0.041	0.036	−0.004	0.003	0.024	−0.001	0.016
SMA	-	-	−0.009	0.003	−0.011	0.027	−0.003	−0.026	0.013	−0.007	−0.001
IMP	0.321 **	0.339 **	−0.018	−0.001	−0.013	−0.027	−0.033	−0.044	−0.018	−0.026	−0.022
**Males**											
IGD	-	0.243 **	−0.041	−0.025	−0.018	0.029	0.013	−0.021	0.000	0.001	0.043
SMA	-	-	−0.030	−0.011	−0.035	−0.009	−0.012	−0.030	0.005	−0.011	0.004
IMP	0.257 **	0.276 **	−0.047	−0.062	−0.081	−0.049	−0.045	−0.059	−0.023	−0.018	−0.004

* *p* < 0.05; ** *p* < 0.01. SMA = Social Media Addiction; IGD = Internet Gaming Disorder; IMP = Impulsiveness; SOM = Somatization; O-C = Obsessive-Compulsive; I-S = Interpersonal Sensitivity; DEP = Depression; ANX = Anxiety; HOS = Hostility; PHOB = Phobic Anxiety; PAR = Paranoid Ideation; PSY = Psychoticism.

**Table 4:** Bivariate correlations between the variables of interest for gender per age groups.

	IGD		SMA	SOM	O_C	I_S	DEP	ANX	HOS	PHOB	PAR	PSY
**Age Group 14–15 Years**												
F	IGD	-	-	0.053	0.062	0.108	0.011	0.011	0.032	0.029	0.013	0.034
	SMA	0.341 **	-	0.016	0.042	0.043	0.026	0.015	0.013	0.034	−0.019	−0.007
	IMP	0.369 **	0.367 **	0.052	0.072	0.097	0.053	0.056	−0.001	0.038	0.023	0.046
M	IGD	-	0.163	0.057	0.067	0.146	0.163	0.131	0.028	0.105	0.073	0.139
	SMA	0.163	-	−0.062	−0.005	−0.011	0.005	−0.032	−0.046	0.042	−0.003	0.006
	IMP	0.196 *	0.350 **	−0.039	−0.034	−0.072	−0.056	−0.062	−0.042	−0.012	−0.052	−0.014
**Age Group 16–17 Years**												
F	IGD	-	-	−0.040	−0.034	−0.036	0.065	−0.076	−0.097	−0.017	−0.035	−0.031
	SMA	0.347 **	-	0.027	0.029	−0.021	0.085	0.038	−0.056	0.033	0.048	0.055
	IMP	0.320 **	0.420 **	0.009	0.001	−0.062	−0.020	−0.027	−0.047	0.004	−0.001	−0.004
M	IGD	-	0.244 **	−0.190 *	−0.224 *	−0.246 **	−0.165	−0.164	−0.165	−0.200 *	−0.140	−0.131
	SMA	0.244 **	-	−0.038	−0.085	−0.101	−0.030	−0.035	−0.079	−0.069	−0.071	−0.044
	IMP	0.291 **	0.239 **	−0.116	−0.162	−0.176	−0.087	−0.077	−0.137	−0.110	−0.062	−0.053
**Age Group 18–19 Years**												
F	IGD	-	-	0.041	0.085	0.046	0.076	0.121	0.152	0.121	0.046	0.085
	SMA	0.184	-	−0.073	−0.075	−0.079	−0.047	−0.054	−0.021	−0.025	−0.046	−0.036
	IMP	0.263 *	0.157	−0.161	−0.134	−0.137	−0.169	−0.191	−0.108	−0.143	−0.132	−0.152
M	IGD	-	-	0.133	0.242	0.143	0.142	0.168	0.159	0.209	0.234	0.213
	SMA	0.411 **	-	0.088	0.165	0.075	0.014	0.103	0.115	0.096	0.164	0.116
	IMP	0.319 *	0.194	0.126	0.139	0.127	0.035	0.059	0.076	0.160	0.167	0.121

* *p* < 0.05; ** *p* < 0.01. F = Females; M = Males; SMA = Social Media Addiction; IGD = Internet Gaming Disorder; IMP = Impulsiveness; SOM = Somatization; O-C = Obsessive-Compulsive; I-S = Interpersonal Sensitivity; DEP = Depression; ANX = Anxiety; HOS = Hostility; PHOB = Phobic Anxiety; PAR = Paranoid Ideation; PSY = Psychoticism.

**Table 5:** Partial correlations between the variables of interest for the total sample.

	SOM	O_C	I_S	DEP	ANX	HOS	PHOB	PAR	PSY
IGD	−0.061	−0.007	0.000	0.071	−0.010	−0.044	0.016	−0.024	0.075
SMA	−0.050	0.018	−0.057	0.054	0.016	−0.056	0.044	−0.008	0.029
IMP	−0.007	0.005	−0.041	−0.008	−0.020	−0.051	0.016	0.023	0.062

SMA = Social Media Addiction; IGD = Internet Gaming Disorder; IMP = Impulsiveness; SOM = Somatization; O-C = Obsessive-Compulsive; I-S = Interpersonal Sensitivity; DEP = Depression; ANX = Anxiety; HOS = Hostility; PHOB = Phobic Anxiety; PAR = Paranoid Ideation; PSY = Psychoticism.

**Table 6:** Partial correlations between the variables of interest for gender groups.

		SOM	O_C	I_S	DEP	ANX	HOS	PHOB	PAR	PSY
	IGD	0.002	0.004	0.075	0.052	−0.078	−0.023	0.018	−0.037	0.025
F	SMA	−0.040	0.012	−0.043	0.080	0.013	−0.057	0.050	−0.017	0.004
	IMP	−0.006	0.069	0.028	−0.028	−0.012	−0.062	−0.019	−0.006	0.009
	IGD	−0.110	0.013	−0.096	0.068	0.059	0.059	0.001	−0.017	0.137 *
M	SMA	−0.062	0.029	0.079	0.025	0.022	−0.050	0.032	0.002	0.062
	IMP	−0.007	−0.058	−0.123 *	0.003	−0.029	−0.031	0.045	0.056	0.116

* *p* < 0.05. SMA = Social Media Addiction; IGD = Internet Gaming Disorder; IMP = Impulsiveness; SOM = Somatization; O-C = Obsessive-Compulsive; I-S = Interpersonal Sensitivity; DEP = Depression; ANX = Anxiety; HOS = Hostility; PHOB = Phobic Anxiety; PAR = Paranoid Ideation; PSY = Psychoticism.

**Table 7:** Partial correlations between the variables of interest for gender per age groups.

		SOM	O_C	I_S	DEP	ANX	HOS	PHOB	PAR	PSY
**Age 14–15**										
F	IGD	0.051	0.034	0.235 **	−0.096	−0.116	0.020	−0.115	0.012	0.030
	SMA	−0.025	0.057	0.024	0.012	0.017	0.008	0.047	−0.066	−0.079
	IMP	−0.037	0.095	0.109	−0.041	0.039	−0.117	−0.085	−0.045	0.059
M	IGD	−0.048	−0.071	0.123	0.159	0.029	−0.182 *	0.051	−0.123	0.122
	SMA	−0.139	0.018	−0.015	0.090	-0.091	−0.083	0.149	0.052	0.097
	IMP	0.000	−0.036	−0.077	−0.070	−0.045	−0.012	0.059	−0.039	0.092
**Age 16–17**										
F	IGD	0.002	−0.021	0.121	0.187 *	−0.114	−0.095	0.069	−0.008	0.059
	SMA	−0.034	0.019	−0.183 *	0.158	0.122	−0.197 *	−0.045	0.079	0.044
	IMP	0.088	0.033	−0.125	−0.015	0.118	−0.061	−0.005	−0.018	0.069
M	IGD	−0.006	−0.090	−0.210 *	0.036	0.051	0.008	−0.038	0.008	0.152
	SMA	0.099	−0.093	−0.175	0.092	0.134	−0.094	0.025	−0.134	0.036
	IMP	0.012	−0.116	−0.249	0.079	−0.034	−0.110	0.029	0.014	0.155
**Age 18–19**										
F	IGD	−0.183	0.056	−0.226	0.027	0.212	0.137	0.198	−0.086	−0.104
	SMA	−0.089	−0.077	−0.094	0.043	−0.001	0.085	0.092	0.044	0.004
	IMP	−0.041	0.070	0.000	−0.055	−0.328 *	0.162	−0.044	0.135	−0.032
M	IGD	−0.317 *	0.210	−0.077	−0.058	−0.162	0.100	0.112	0.184	0.071
	SMA	0.112	0.151	−0.116	−0.323 *	0.001	0.088	−0.140	0.209	0.136
	IMP	−0.082	−0.014	0.143	−0.125	−0.199	0.115	0.213	0.288 *	−0.081

* *p* < 0.05; ** *p* < 0.01. F = Females; M = Males; SMA = Social Media Addiction; IGD = Internet Gaming Disorder; IMP = Impulsiveness; SOM = Somatization; O-C = Obsessive-Compulsive; I-S = Interpersonal Sensitivity; DEP = Depression; ANX = Anxiety; HOS = Hostility; PHOB = Phobic Anxiety; PAR = Paranoid Ideation; PSY = Psychoticism.
